# Integrative Multi-Omics Characterization and Structural Insights into the Poorly Annotated Integrin *ITGA6* X1X2 Isoform in Mammals

**DOI:** 10.3390/genes16101134

**Published:** 2025-09-25

**Authors:** Ximena Aixa Castro Naser, Alessandro Cestaro, Silvio C. E. Tosatto, Emanuela Leonardi

**Affiliations:** 1 Department of Biomedical Sciences, University of Padua, 35131 Padua, Italy; 2 Institute of Biomembranes, Bioenergetics and Molecular Biotechnologies, National Research Council (CNR-IBIOM), 70126 Bari, Italy; 3 Fondazione Edmund Mach (FEM), 38098 San Michele All’Adige, Italy

**Keywords:** genome annotation, integrins, ITGA, alternative splicing

## Abstract

Background: Accurate annotation of gene isoforms remains one of the major obstacles in translating genomic data into meaningful biological insight. Laminin-binding integrins, particularly integrin α6 (*ITGA6*), exemplify this challenge through their complex splicing patterns. The rare *ITGA6* X1X2 isoform, generated by the alternative inclusion of exons X1 and X2 within the β-propeller domain, has remained poorly characterized despite decades of integrin research. Methods: We combined comparative genomics across primates with targeted re-alignment to assess exon conservation and annotation fidelity; analyzed RNA-seq for exon-level usage; applied splice-site prediction to evaluate inclusion potential; surveyed cancer mutation resources for exon-specific variants; and used structural/disorder modeling to infer effects on the β-propeller. Results: Exon X2 is conserved at the genomic level but inconsistently annotated, reflecting the limitations of current annotation pipelines rather than genuine evolutionary loss. RNA-seq analyses reveal low but detectable expression of X2, consistent with weak splice site predictions that suggest strict regulatory control and condition-specific expression. Despite its rarity, recurrent mutations in exon X2 are reported in cancer datasets, implying possible roles in disease. Structural modeling further indicates that X2 contributes to a flexible, disordered region within the β-propeller domain, potentially influencing laminin binding or β-subunit dimerization. Conclusions: Altogether, our results suggest that *ITGA6* X1X2 could be a rare, tightly regulated isoform with potential functional and pathological relevance.

## 1. Introduction

Since the first human genome was published [1,2], rapid advances in sequencing and reduced costs have enabled the sequencing of thousands of organisms [3,4]. Public repositories now host over 35,000 eukaryotic genomes in Ensembl [5] and 17,823 in GenBank [6], thereby greatly advancing evolution and gene function.

However, genome annotation remains a major challenge, as even the human genome continues to undergo revisions [7]. Annotations in non-model organisms are often incomplete, limiting cross-species analyses [8,9]. A key issue is alternative splicing, which produces multiple protein isoforms by selectively including or excluding specific exons [10]. Despite being common in humans, most isoforms remain undetected at the protein level due to mass spectrometry and peptide reference libraries limitations [11,12].

Integrins are transmembrane receptors mediating cell–cell and cell–extracellular matrix interactions. Mammals express 18 α and 8 β subunits, forming 24 heterodimers with distinct ligand specificities and cellular functions [13,14]. Laminin-binding integrins (α3β1, α6β1, α6β4, and α7β1) are crucial for epithelial integrity, tissue architecture, and signaling [15,16]. Compared with other integrin subfamilies, they are less studied, yet they possess unique functions as the only receptors capable of linking cells to laminins. Through this interaction, they regulate processes such as angiogenesis, embryonic development, cell polarity, and migration [17,18,19,20]. Because of this, they are implicated in various diseases [21,22] including cancer [23,24]. Despite their therapeutic potential, no drug targeting these integrins has reached clinical trials [25].

The functional complexity of laminin-binding integrins is increased by alternative splicing of the α3, α6, and α7 subunit mRNAs, producing isoforms with distinct extracellular and/or intracellular domains that affect ligand binding and signaling [26]. Integrin α3 has two cytoplasmic variants (A and B) [27]. Integrins α6 and α7 have greater diversity, differing in both their cytoplasmic (A and B) and extracellular domains (X1 and X2) [28,29,30]. These isoforms display tissue- and stage-specific expression, but their biological roles remain unclear. For α6, the cytoplasmic isoforms are the best characterized [29,31,32] and two extracellular forms exist, one of which includes the alternative incorporation of exons X1 and X2 within the β-propeller domain. Despite RNA and proteomics evidence [28], the integrin α6X1X2 isoform is poorly understood, with unknown regulatory, structural, and functional features.

In this study, we investigated the annotation, expression, and evolutionary conservation of the integrin α6X1X2 using genomics, transcriptomics, and proteomics data, highlighting broader challenges in genome annotation and the study of alternative splicing.

To distinguish between gene and protein references throughout this study, we follow standard nomenclature: Gene and transcript names are indicated in uppercase letters (e.g., integrin alpha-6 (*ITGA6*)), while protein products are referred to using the corresponding Greek letter notation (e.g., α6).

## 2. Materials and Methods

### 2.1. Sequence Retrieval, Alignment, and Phylogenetic Analysis

Protein sequences of integrins and their isoforms were retrieved from the UniProt database (accession date: 3 December 2024) [33], and transcript/genomic sequences from RefSeq [34] and Ensembl [5]. Multiple sequence alignments were calculated using ClustalW 2.1 [35] with default parameters and manually edited in Jalview 2.11.4.1 [36]. A phylogenetic tree was generated using W-IQ-TREE web server [37] with the JTT+G substitution model, automatically selected based on the Bayesian information criterion (BIC), and branch support assessed using 1000 ultrafast bootstrap replicates. The resulting tree was visualized in iTOL v7 [38].

### 2.2. Genome Annotation Assessment and Splicing Prediction

Genome Threader v1.7.3 [39] was used to assess genome annotation of *Macaca mulatta* *ITGA6*, inputting the full genomic sequence along with *ITGA6* X1 mRNAs (RefSeq accession: NM_001258117.1 and XM_015110360.2) from *M. mulatta*, *ITGA6* X1X2 cDNA (Ensembl accession: ENSMFAT00000012408.2) and protein (UniProt accession: A0A2K5WLV0) from *Macaca fascicularis*; *ITGA6* X1X2 cDNA (Ensembl accession: ENST00000442250.6) and protein (UniProt accession: P23229-1) from *Homo sapiens*; as well as *ITGA6* X1 cDNA (Ensembl accession: ENST00000684293.1) from *H. sapiens*.

RNAseq data for *M. mulatta* were obtained from the NCBI Sequence Read Archive (SRA) using the Run Selector tool [6]. From 51,814 experiments, 63 experiments labeled as heart and kidney tissues were selected to analyze exon presence. Reads were aligned to chromosome 12 of *M. mulatta* using MagicBLAST v1.7.2 with parameters by default [40] and alignments visualized with IGV v2.19.1 [41] to confirm exon usage and splicing events.

Splice site predictions, including branch points and auxiliary sequences of exons X1 and X2, were performed using Human Splicing Finder (HSF) [42], with a ±20 bp flanking region and a 30 bp window size for GC content estimation. Splice site strength was evaluated using the HSF matrix (range: 65 = weak to 100 = very strong) and the MaxEnt matrix (range: 3 = weak to 12 = very strong). For cross validation, we used SpliceRover [43] with default parameters.

### 2.3. Expression, Variant, and Mutation Analysis

Transcript expression was analyzed using the GTEx v10 Portal [44] of RNA-seq from healthy individuals. Proteomic data were obtained from ProteomicsDB [45] and PeptideAtlas [46] (accession date: 18 February 2025). Cancer-associated mutations were queried in COSMIC v101 [47]. Rare variants affecting the human *ITGA6* X1X2 transcript (RefSeq accession: NM_001394928.1, and Ensembl accession: ENST00000442250.6) for exons X1 and X2 were examined using Ensembl113 [5], gnomAD v2.1.1 and v4.1.0 [48], and ClinVar [49] (accession date: 27 February 2025), annotating their frequency, consequence, and clinical significance.

### 2.4. Structural Modeling and Contact Analysis

Secondary structure predictions were performed using Fast Estimator of Latent Local Structure (FELLS) v1.1 [50]. To assess integrin structure, we used both experimental and predictive models. The experimental structure of the integrin α6β1 dimer in complex with laminin-511 (PDB code: 7CEC) [51] was obtained from the Protein Data Bank [52]. In addition, we generated protein structure models of the α6X1X2β1 integrin using AlphaFold 3 [53], based on the full-length protein sequence including both alternatively spliced exons. Model confidence was evaluated using pLDDT scores. All structures were visualized, aligned, and compared using PyMOL 2.5 [54].

Wild-type (WT) α6X1X2β1–laminin511 complexes (five AlphaFold models) and variant models (p.E233Q, p.R218H) were analyzed in RING v4.0 [55]. Contacts were calculated with strict thresholds: hydrogen-bond donor–acceptor ≤3.9 Å, H–acceptor ≤2.5 Å, ionic (salt-bridge) ≤4.0 Å, π–π stacking (ring centers) ≤6.5 Å, cation–π ≤5.0 Å, π–H donor-to-ring center ≤4.3 Å, metal-ion coordination ≤2.8 Å, disulfide S–S ≤2.5 Å; van der Waals contacts radii-intersection fraction ≥0.01. Occupancy was reported as n/5 models (WT and variants).

## 3. Results

### 3.1. Annotation Quality of α Integrins Across Mammals

**Figure 1:**
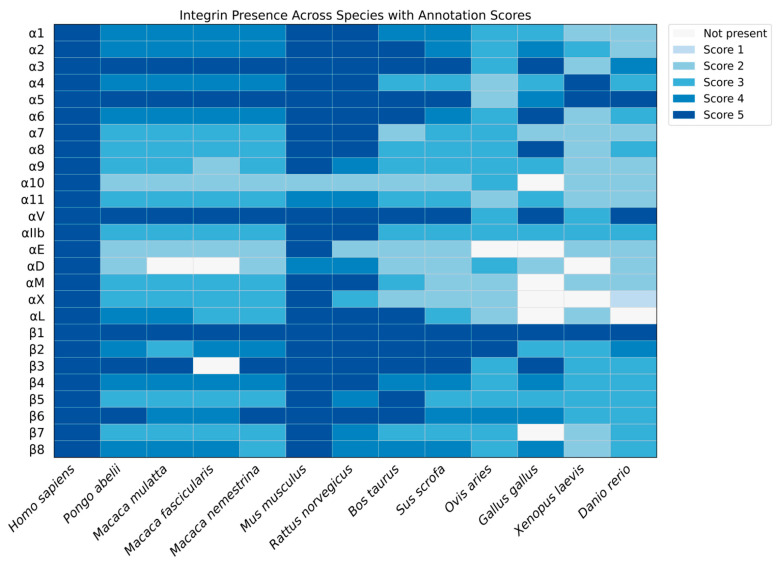
Heatmap showing the presence of integrin proteins across various species. Cell color represents the UniProt annotation score for each protein and species, with dark blue representing well-annotated entries (score 5); progressively lighter blue colors are used for lower scores. White cells indicate absence of the protein in the database.

Analysis of mammalian α-integrin annotations revealed widespread inconsistency. Humans have complete, high-quality annotations for all 18 genes, and mice for 17 (lacking α10), but most species lack reliable data for most genes, limiting comparative analyses of integrin gene families (Figure 1).

**Figure 2:**
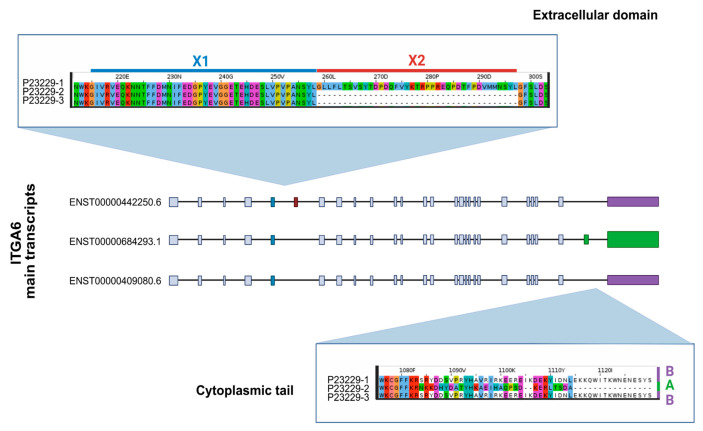
Schematic representation of the main *ITGA6* transcripts (Ensembl). Exons are shown as boxes and introns as connecting lines. Colored boxes indicate variable regions in the extracellular domain (X1, blue; X2, red) and cytoplasmic tail (A, green; B, purple). Corresponding UniProt protein isoforms are shown in zoomed callouts. Residues are colored based on the Clustal colour scheme.

We focused on laminin-binding integrins due to their unique roles in basement membrane organization and their extensive isoform diversity generated by alternative splicing, despite being a less-studied subgroup compared to other integrin families. Among them, *ITGA6* is particularly notable for its ability to pair with both β1 and β4 subunits and for its complex alternative splicing in both the extracellular (X1 and X2) and intracellular (A and B) domains. The human *ITGA6* gene (chromosome 2: 172,234,216–172,506,459) has several Ensembl transcripts, five matching reviewed UniProt proteins (P23229-1, -2, -3, -5, -7), and two RefSeq entries (NM_001394928.1 and NM_000210.4). Key isoforms include *ITGA6* X1A (Matched Annotation from NCBI and EMBL-EBI (MANE) Select, Ensembl Canonical: Ensembl ID: ENST00000684293.1, RefSeq accession: NM_000210.4, UniProt accession: P23229-2), and *ITGA6* X1X2B (MANE Plus Clinical, UniProt Canonical: Ensembl ID: ENST00000442250.6, RefSeq accession: NM_001394928.1, UniProt accession: P23229-1) (Figure 2).

### 3.2. Presence of the ITGA6X1X2 Isoform Across Species

**Figure 3:**
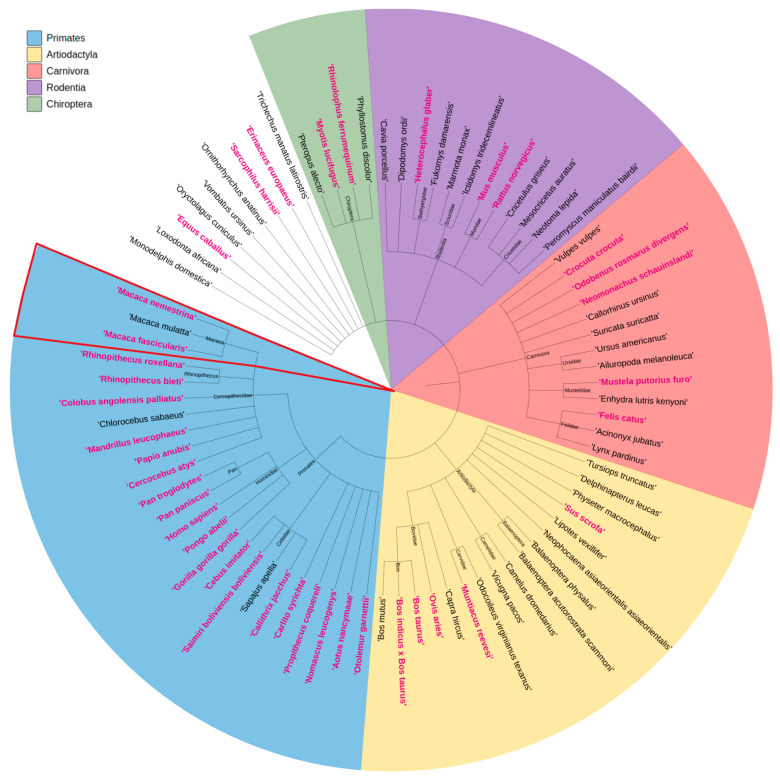
Circular phylogenetic tree showing selected mammalian species. Clades are colored by taxonomic order: Primates (blue), Artiodactyla (yellow), Carnivora (red), Rodentia (purple), and Chiroptera (green). Species names in pink have at least one *ITGA6* isoform containing both X1 and X2 exons. Species names in black lack isoforms with the X2 exon. Example highlighted: *Macaca nemestrina* and *Macaca fascicularis* represent primates with X1X2 isoforms, while *Macaca mulatta* has no X1X2 isoform annotated.

We examined *ITGA6* X1X2 distribution across species (Figure 3). Using Ensembl and UniProt data, we found no clear evolutionary pattern, suggesting a seemingly random presence of the X1X2 variant. As an illustrative example of this inconsistency, we focused on three *Macaca* species. Although closely related, *M. nemestrina* and *M. fascicularis* have transcripts annotated with both the X1-only and X1X2 isoforms, whereas *M. mulatta* has only the X1 exon represented in current transcript models (see Figure S1). This highlights how annotation inconsistencies, even among phylogenetically close species, can complicate comparative analyses.

In *Macaca mulatta*, exon X2 was absent from all annotated isoforms, yet a BLAST search showed 100% identity with a genomic region in intron 5 of the *ITGA6* gene, suggesting an annotation omission. Using GenomeThreader with RNA, cDNAs, and protein sequences from *M. mulatta, human*, and *M. fascicularis,* we predicted full transcripts including the X2 exon. Its inclusion preserved the reading frame, indicating functional splicing sites.

RNA-seq data of 63 heart or kidney datasets—tissues expressing *ITGA6* X1X2 in humans [28]—revealed 16 reads mapping to X2 with full coverage and junction-spanning reads (Table 1). Although low counts limit conclusions, the presence of spliced reads supports the idea that *ITGA6* X1X2 transcripts may be expressed in other species even when not represented in current annotations. This is not unexpected, as low abundance isoforms are easily missed by fully automated annotation pipelines and often require manual curation or dedicated gene models.

**Table 1:** RNA-seq reads from *Macaca mulatta* aligned to the full chromosome 12 within the region of exon X2. The table lists the SRA experiment identifiers; the representative read names and the genomic reference spans to which they map.

Experiment	Read Name	Reference Span
ERX2613198	ERR2596913.20999958	NC_041765.1:59,585,017–59,585,117
ERX2613229	ERR2596944.16351931	NC_041765.1:59,585,019–59,585,119
ERX2613087	ERR2596802.8153246	NC_041765.1:59,585,062–59,585,162
ERX2613214	ERR2596929.22893645	NC_041765.1:59,585,134–59,585,234
ERX2613201	ERR2596916.15064411	NC_041765.1:59,585,177–59,585,277
ERX2613119	ERR2596834.21730323	NC_041765.1:59,585,181–59,585,281
ERX2613236	ERR2596951.7152511	NC_041765.1:59,583,397–59,585,162
ERX2613177	ERR2596892.11271980	NC_041765.1:59,585,192–59,585,290
ERX2613199	ERR2596914.34156819	NC_041765.1:59,585,063–59,585,161
ERX2613116	ERR2596831.72595928	NC_041765.1:59,585,068–59,585,167
ERX2613191	ERR2596906.34031824	NC_041765.1:59,585,111–59,585,210
ERX2613229	ERR2596944.50503340	NC_041765.1:59,585,113–59,585,213
ERX2613153	ERR2596868.17044807	NC_041765.1:59,585,069–59,585,169
ERX2613217	ERR2596932.28822930	NC_041765.1:59,585,074–59,585,174
ERX2613171	ERR2596886.27577682	NC_041765.1:59,585,077–59,585,176
ERX2613159	ERR2596874.8771744	NC_041765.1:59,583,353–59,585,118

### 3.3. Splicing Signal Analysis for Exons X1 and X2

We evaluated the splicing potential of *ITGA6* and *ITGA7* exons X1 and X2 using Human Splicing Finder (HSF), MaxEntScan (Figure S2) [42], and SpliceRover (Figure S3) [43]. In *ITGA6*, HSF scores were high for X1 (acceptor: 84.77/donor: 88.63) and slightly lower for X2 (79.15/85.11); MaxEnt scores were 11.14/7.00 for X1 and much lower for X2 (4.92/4.07). In *ITGA7*, X1 HSF scores were 89.76/82.95 (MaxEnt 7.47/4.44) and X2 scores 80.94/85.11 (MaxEnt 9.32/4.07). SpliceRover showed strong X1 signals (0.98/0.71) but weak X2 acceptor in *ITGA6* (0.20/0.80). Overall, *ITGA6* X2 has weaker splice signals, especially at the acceptor site, potentially limiting inclusion without specific regulatory factors.

### 3.4. Transcript and Protein Evidence in Humans

**Figure 4:**
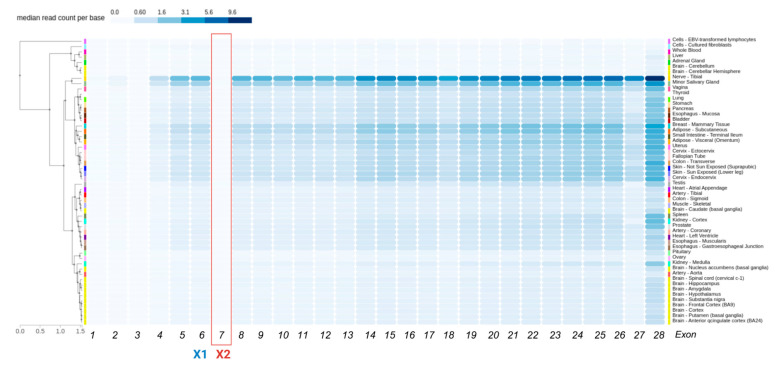
GTEx exon-level expression profile of human *ITGA6*. Heatmap shows median read count per base for each *ITGA6* exon across GTEx tissue samples, clustered by expression pattern. Tissue types are color-coded at left. Exons X1 (blue) and X2 (red) are highlighted.

Data showed *ITGA6* X2 exon expression was nearly undetectable across tissues, based on median read count per base as reported by GTEx (Nerve-Tibial 0.0171, Minor Salivary Gland 0.00855, Testis 0.00855, Uterus 0.00472, Vagina 0.00362, Breast 0.000112) (Figure 4). In contrast, *ITGA7*, X1, and X2 isoforms show robust, tissue-specific expression: X2 in Skeletal Muscle (3.08), Heart-Left Ventricle (2.77), Heart-Atrial Appendage (3.21); X1 in Bladder (1.90), Fallopian Tube (2.32), Colon-Sigmoid (2.69), Esophagus-Muscularis (2.98), Esophagus-Gastroesophageal Junction (2.64), Artery-Aorta (3.97), Artery-Tibial (5.15), Artery-Coronary (3.24).

ProteomicsDB reported 73.19% coverage and 117 unique peptides for *ITGA6* X1X2, but none for X2; *ITGA7* had 57.92% coverage with peptides for X2 (Figure S4). PeptideAtlas detected two peptides from *ITGA6* X2 (one and two observations), though the region is flagged “Unlikely due to SSR” and “Unlikely due to Length,” explaining low detection (Figure S5).

### 3.5. Variant Distribution and Mutations in Human Exons X1 and X2

In *Homo sapiens* *ITGA6*, Ensembl lists 15 X1 variants (one likely pathogenic, three uncertain, and 11 likely benign) and four in X2 (three likely benign and one benign). gnomAD reports 13 X1 variants (one likely pathogenic, three uncertain, and nine likely benign) and two in X2 (one likely benign and one benign) (Table S1). ClinVar annotates 10 X1 variants (one likely pathogenic, two uncertain—linked to epidermolysis bullosa with pyloric atresia and other genetic diseases, and seven likely benign) and two X2 variants (one benign and one likely benign) (Table S1). As context, gnomAD v4.1.0 shows that *ITGA6* (Ensembl ID: ENST00000442250.6) has moderate missense depletion and borderline loss-of-function (LoF) intolerance (missense Z = 2.47; LOEUF = 0.61). We also used gnomAD v2.1.1 to assess regional missense constraints in *ITGA6*; no exons showed a significant deviation in the missense observed/expected ratio.

The analysis of COSMIC histology annotations revealed that *ITGA6* X1 mutations are distributed across multiple epithelial cancer types, including urinary tract, breast, lung, stomach, and large intestine carcinomas, as well as several melanomas. In contrast, *ITGA6* X2 variants are less frequent but more represented in skin-associated malignancies, such as basal cell carcinoma, squamous cell carcinoma, and melanoma (Table S2), suggesting a possible link to basement membrane-related processes. We identified notable amino acid substitutions in *ITGA6* X1 (p.Q221*, p.K222N, p.E238A, and p.E247*) and X2 (p.S267F, p.P281T, p.P281L, and p.V291L) across multiple tissues, plus high copy number gains. Rare events include a single insertion in X1 (p.D228*) and a deletion in X2 (p.L297*). *ITGA7* X1 showed higher mutation density, with up to seven variants at p.R257: p.R257C (*n* = 3), p.R257H (*n* = 2), p.R257S (*n* = 1), and p.R257 = (*n* = 1)) (see Table S2). Normalizing by exon length, *ITGA6* X2 showed a mutation density of ~75.8 variants/kb, comparable to *ITGA7* X2 (~75.0 variants/kb) but lower than *ITGA6* X1 (~121.2 variants/kb) and *ITGA7* X1 (~205.1 variants/kb) (Table S3). These findings indicate that *ITGA6* X2 is not unusually protected from somatic mutation relative to other laminin-binding integrins, but its higher representation in skin-associated cancers highlights a potential tissue-specific relevance. However, the functional impact remains uncertain and requires experimental validation.

### 3.6. Structural Analysis

**Figure 5:**
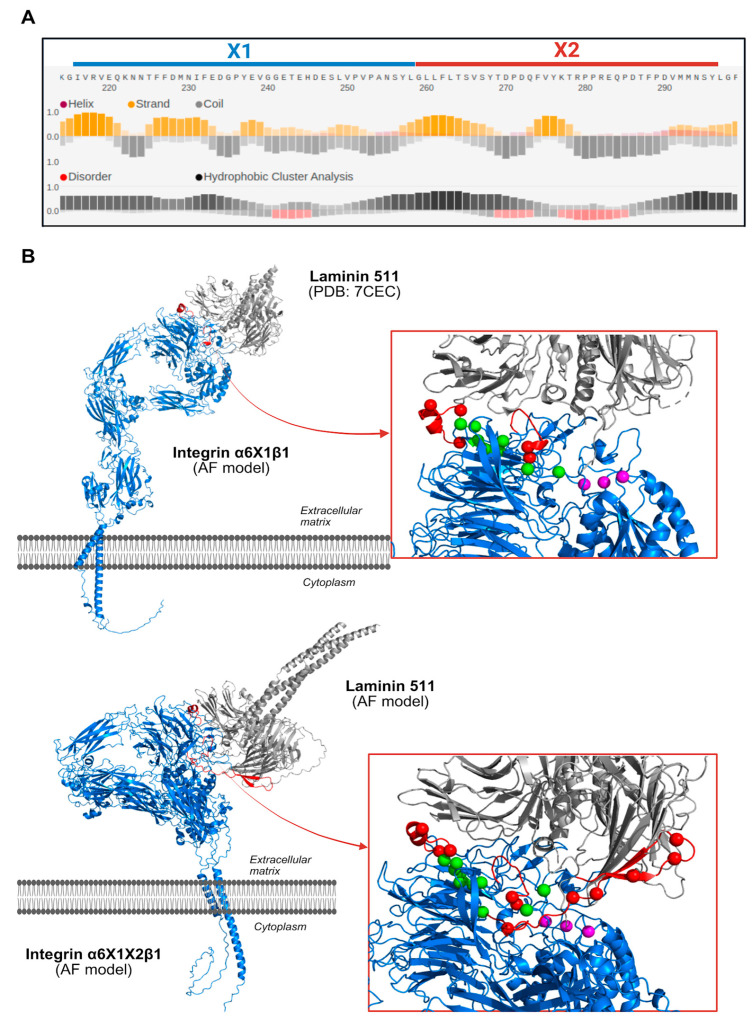
(**A**) FELLS secondary structure prediction for integrin α6 exons X1 and X2. Predicted secondary structure and disorder propensity are shown for residues spanning exons X1 (blue) and X2 (red). Most residues exhibit strong coil propensity and β strands. Multiple residues in both exons are predicted to be disordered. (**B**) Structural models of α6β1 integrin in complex with laminin-511. The integrin α6β1 dimer is shown in blue, laminin-511 in grey, and the X1 and X2-encoded segments in red. Insets highlight the laminin-binding interface, with missense mutations from COSMIC represented as spheres: Red spheres indicate variants located in disordered regions, whereas green spheres represent variants in structured regions. Variants are mapped at positions 218, 219, 220, 222, 230, 233, 234, 235, 238, 249, 250, 251, and 254 (X1) and 263, 267, 271, 279, 281, 291, and 298 (X2). The SyMBS, MIDAS, and ADMIDAS metal-binding sites are shown as magenta spheres. Top: experimental cryo-EM structure of α6X1β1–laminin-511 (PDB code: 7CEC), with the AlphaFold 3 model of X1 disordered loops (red). Bottom: AlphaFold 3 model of α6X1X2β1 bound to laminin-511. In the α6X1β1 complex, the region corresponding to X1 forms short loops in the β-propeller domain, whereas in the α6X1X2β1 model, the X2-encoded segment is fully disordered, adopting unstructured loops and occasional β-strand-like conformations near the binding site.

FELLS secondary structure prediction showed that most residues in exons X1 and X2 have strong coil propensity, with fewer adopting β-strand conformations. Several residues in both exons were identified as disordered regions, indicating high flexibility (see Figure 5A).

For the three-dimensional structural analysis, the experimental structure of the α6X1β1–laminin-511 complex (PDB: 7CEC) was used. This structure captures the β-propeller region of α6 bound to the β domain of integrin β1, forming the laminin-binding interface, but it lacks some residues corresponding to exon X1. To address this, the heterodimer was modeled using AlphaFold 3, which provided a complete structure of the integrin dimer in an extended conformation. In the α6 subunit, the model shows the FG-GAP β propeller with seven blades, followed by three immunoglobulin-like domains (Thigh, CALF-1, and CALF-2) in the extracellular region, and the transmembrane helix with a disordered cytoplasmic tail. The residues missing in the PDB structure were predicted as disordered loops within the β-propeller region, consistent with previous evidence (Figure 5B).

To explore the structural effects of exon X2 inclusion, we modeled the full α6X1X2β1–laminin-511 complex using AlphaFold 3 and compared it to the experimental structure of the α6X1β1–laminin-511 complex (PDB code: 7CEC). The overall architecture of the heterodimer was preserved, with the β-propeller, thigh, CALF domains, transmembrane, and cytoplasmic regions all clearly resolved. However, the α6X1X2β1 complex adopted a more bent conformation relative to the α6X1 complex, while still maintaining the same global domain organization. The AlphaFold models also revealed the X2-encoded region as fully disordered, alternating between unstructured loops and transient β-strand-like shapes near the laminin-binding interface (Figure 5B). This contrasts with the predominantly well-structured β-propeller domain in the crystallized α6X1β1 integrin, suggesting X2 introduces notable structural flexibility, and it could transiently interact with laminin, alter the local binding environment, or affect the heterodimer stability.

Several missense variants identified in COSMIC were located within the ligand-binding surface of the α6X1X2 region. For example, we observed substitutions that neutralize negatively charged residues, such as p.E220Q, p.E233Q, p.D234N, and p.E238A, as well as changes that remove or replace positively charged residues, including p.R218H, p.K222N, and p.R279Q. These charge-altering variants may disrupt the local electrostatic environment of the β-propeller domain, which is critical for mediating integrin–laminin interactions.

**Figure 6:**
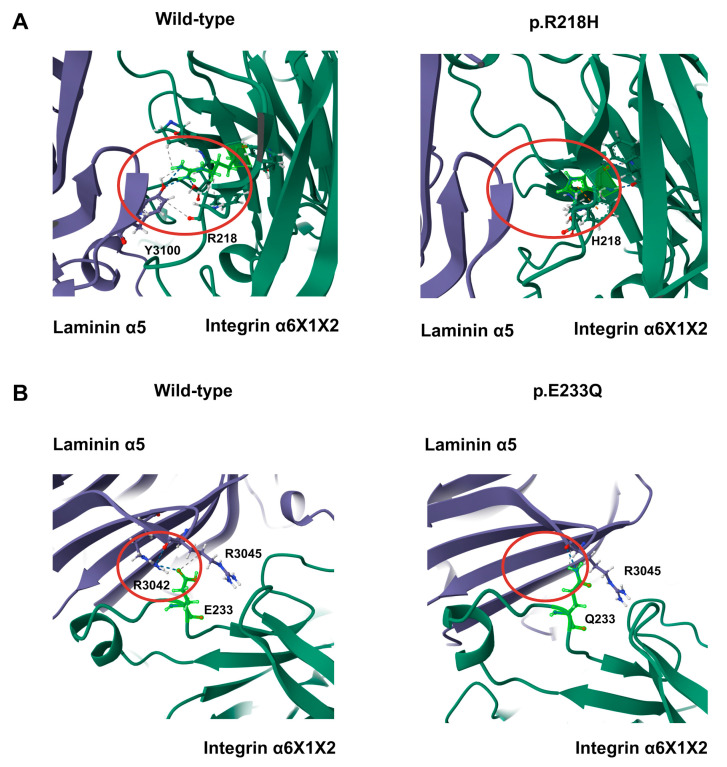
Cancer-associated *ITGA6* variants reduce laminin contacts at the β-propeller interface. Cartoon view of the α6X1X2β1–laminin-511 complex. Integrin α6X1X2 is shown in green, laminin-α5 in purple; the mutated α6 residue and the contacting laminin residue are shown as sticks. Red circles mark the interaction region. (**A**) Wild-type (**left**) versus p.R218H (**right**). In WT, R218 frequently forms a hydrogen bond with laminin-α5 Y3100 (contact present in 3/5 WT models); the interaction is absent in R218H (0/5). (**B**) Wild-type (**left**) versus p.E233Q (**right**). In WT, E233 forms a recurrent hydrogen bond with laminin-α5 R3042 (5/5 WT models). The E233Q substitution abolishes the H-bond; only van der Waals proximity is observed in 3/5 models and no contact in 2/5.

To test this hypothesis, we modeled representative variants in the α6X1X2β1–laminin-511 complex and compared them with an AlphaFold WT ensemble (*n* = 5). RING contact analysis showed that, in WT, E233 forms a hydrogen bond (H-bond) with laminin-α5 R3042 in 5/5 models; in E233Q, this interaction was abolished (H-bond 0/5), with only van der Waals (VDW) proximity in 3/5 models and no contact in 2/5 (Figure 6B). Likewise, R218 forms a hydrogen bond to laminin-α5 Y3100 in 3/5 WT models, whereas R218H shows no contact in any model (0/5) (Figure 6A).

While experimental validation will be required, these analyses indicate that charge-altering substitutions at the β-propeller surface reduce the local electrostatic complementarity and the probability of stabilizing H-bond/VDW contacts with laminin.

## 4. Discussion

This study examined the annotation, conservation, and expression of the laminin-binding integrin *ITGA6*, focusing on the poorly characterized X1X2 isoform. By integrating transcriptomics, proteomics, and genomics data, we explored the regulatory and functional roles of this alternatively spliced form.

Comparative analysis of the integrin ɑ-subunit family across mammals revealed a major issue: inconsistent genome and proteome annotations. Humans and mice have relatively high-quality data, but most mammals lack complete annotation for all 18 ɑ integrins. This hinders studies on gene families and evolutionary conservation, as biologically relevant variants may be missing or misinterpreted. This observation reflects broader concerns that gene prediction inaccuracies and incomplete curation can lead to the misinterpretation of gene or isoform presence or absence in cross-species studies, resulting in falsely inferred gains or losses [56,57,58].

The *ITGA6* X1X2 isoform, generated by splicing of exons X1 and X2 in the β-propeller domain, highlights the challenges of isoform annotation. X1 is consistently represented in databases, but X2 is detected only in a rare, low-abundance transcript co-expressed with X1 [28]. Detection of this isoform varies across species, reflecting annotation gaps rather than true evolutionary divergence. In *Macaca mulatta*, for instance, exon X2 is perfectly conserved in the genome yet absent from annotated transcripts; however, GenomeThreader and comparative alignments predicted possible splice sites, and low-coverage RNA-seq provided junction-spanning reads, suggesting condition-specific transcription. Splice site prediction tools (MaxEntScan, HSF, and SpliceRover) further indicate that X2 has weaker signals, especially at the acceptor site, which likely constrain its inclusion to specific regulatory contexts. Importantly, recent work on the exon-mediated activation of transcription starts shows that even rare alternative exons can modulate promoter usage and gene expression [59], raising the possibility that *ITGA6* X2 contributes regulatory functions beyond its coding capacity.

GTEx data showed near-zero exon X2 expression in most human tissues. ProteomicsDB detected no peptides from the X2 region, while PeptideAtlas reported some, possibly due to database differences in data curation, sample origin, and detection threshold. It is known that mass spectrometry underrepresents short or low-complexity peptides and incompletely captures proteoforms generated by alternative splicing [60]. In contrast, both the X1 and X2 isoforms of *ITGA7* are strongly expressed and easily detected, indicating a more stable splicing mechanism.

Alternative splicing generates transcript and protein diversity and is influenced by environmental cues, stress, and development, while aberrant splicing is linked to diseases such as cancer and neurodegeneration [61,62,63,64]. The α6X1X2 isoform’s limited, co-regulated expression with the ubiquitous α6X1 suggests a tightly controlled, context-dependent role. In *ITGA7*, X1 and X2 isoforms have distinct tissue distributions and activation modes, a regulatory logic that may also apply to *ITGA6*, where X2 inclusion could affect integrin activity or ligand specificity.

Fewer reported variants in exon X2 likely reflect its low expression and limited sequencing coverage. However, COSMIC data show both X1 and X2 exons mutated in cancers, with recurrent X2 mutations despite scarce transcriptomic or proteomic evidence, pointing to possible X2 expression in cancer. However, whether these mutations have functional consequences remains unknown and requires further study.

Both X1 and X2 map to the β-propeller domain, key for ligand binding in laminin-binding integrins. In *ITGA7*, the mutually exclusive splicing of X1 and X2 affects laminin affinity, with X2 favoring laminin-111 and X1 laminins-411 and -511/-521 [65,66]. In *ITGA6*, however, X1 and X1X2 isoforms show similar ligand specificity and require β4 co-expression for α6X1X2β1 formation, suggesting X1X2 may preferentially pair with β4 [28]. Although our study did not assess integrin pairing experimentally, this hypothesis could explain the low proteomic detectability of X1X2 in tissues where β4 expression is low or absent. Further structural and functional studies on X2 inclusion are needed.

Secondary structure predictions and AlphaFold models indicate that exons X1 and X2 encode intrinsically disordered regions, common targets of alternative splicing enriched in motifs and modification sites [67,68,69]. These flexible regions enable the context-specific modulation of protein function, especially in signaling proteins like integrins. In support of this, Arimori et al. [51] hypothesized that the exon X1′s disordered loop may aid ligand capture, and exon X2 likely plays a similar role, modulating ligand interactions or acting as a flexible spacer, as suggested by its variable structure in AlphaFold models depending on laminin contact. The inconsistent annotation and detection of *ITGA6* X1X2 across species likely result from weak splicing signals, low expression, and incomplete genome annotation, rather than true evolutionary loss.

Taken together, our findings position *ITGA6* X1X2 as a rare but potentially functionally relevant isoform that has been overlooked due to annotation gaps, limited transcriptomic and proteomic data, and the inherent detection limits of large-scale methods. Multiple independent but individually modest observations (ranging from splicing signal predictions and RNA-seq data to comparative annotation, somatic variant mapping, and structural modeling) converge to support its biological relevance. The low abundance and inconsistent annotation of this isoform do not diminish its potential importance; rather, they highlight the complexity of identifying condition-specific transcripts within automated pipelines. Improving integrative analysis frameworks and incorporating manual curation will be essential not only for uncovering the full regulatory potential of *ITGA6* but also for accurately characterizing other gene families where rare isoforms may play significant and underappreciated roles.

## 5. Conclusions

Our study highlights an example of the gap between biological complexity and its representation in databases with *ITGA6* X1X2. The inconsistent annotation and detection across species likely result from weak splicing signals, low expression, and incomplete genome annotation, rather than true evolutionary loss. Addressing these issues requires deeper tissue-specific transcriptomic profiling, experimental validation by RT-PCR and proteomics, and targeted reannotation of underrepresented isoforms. More broadly, our work underscores the need to integrate genomics, transcriptomics, and proteomics data to fully capture regulatory and functional diversity in complex gene families like integrins.

## Data Availability

The original contributions presented in this study are included in the article/Supplementary Material. Further inquiries can be directed to the corresponding authors.

## Supplementary Materials

The following supporting information can be downloaded at: https://www.mdpi.com/article/10.3390/genes16101134/s1, Figure S1: Nucleotide and protein sequence alignments of *ITGA6* exons X1 and X2 in macaque species; Figure S2: Human Splice Finder analysis of *ITGA6* and *ITGA7* X1 and X2 exons; Figure S3: SpliceRover results of human *ITGA6*; Figure S4: ProteomicsDB statistics and sequence coverage maps for human integrin α6 and integrin α7; Figure S5: PeptideAtlas protein coverage map for integrin α6; Table S1: List of reported variants within *ITGA6* exons X1 and X2; Table S2: List of reported mutations within *ITGA6* exons X1 and X2 from COSMIC; Table S3: Length-normalized somatic variant burden in alternative exons X1/X2 of *ITGA6* and *ITGA7*.
