# Supplementary material for: Integrative Multi-Omics Characterization and Structural Insights into the Poorly Annotated Integrin ITGA6 X1X2 Isoform in Mammals
Source: Genes (Basel). 2025 Sep 25;16(10):1134. doi: 10.3390/genes16101134 (PMC12564407; doi:10.3390/genes16101134)
Supplement: Supplementary file 1 [file genes-16-01134-s001.zip › Supplementary Figures.pdf]

Integrative Multi-Omics Characterization and Structural Insights into the Poorly Annotated Integrin ITGA6 X1X2 Isoform in Mammals

Ximena Aixa Castro Naser[1], Alessandro Cestaro[2,3], Silvio C. E. Tosatto[1,2,*], Emanuela Leonardi[1,*]

[1] - Department of Biomedical Sciences, University of Padua, 35131 Padua, Italy
[2] - Institute of Biomembranes, Bioenergetics and Molecular Biotechnologies, National Research Council (CNR-IBIOM), 70126 Bari, Italy
[3] - Fondazione Edmund Mach (FEM), 38098 San Michele All'Adige, Italy
* - To whom correspondence should be addressed at: silvio.tosatto@unipd.it; emanuela.leonardi@unipd.it

Supplementary figures

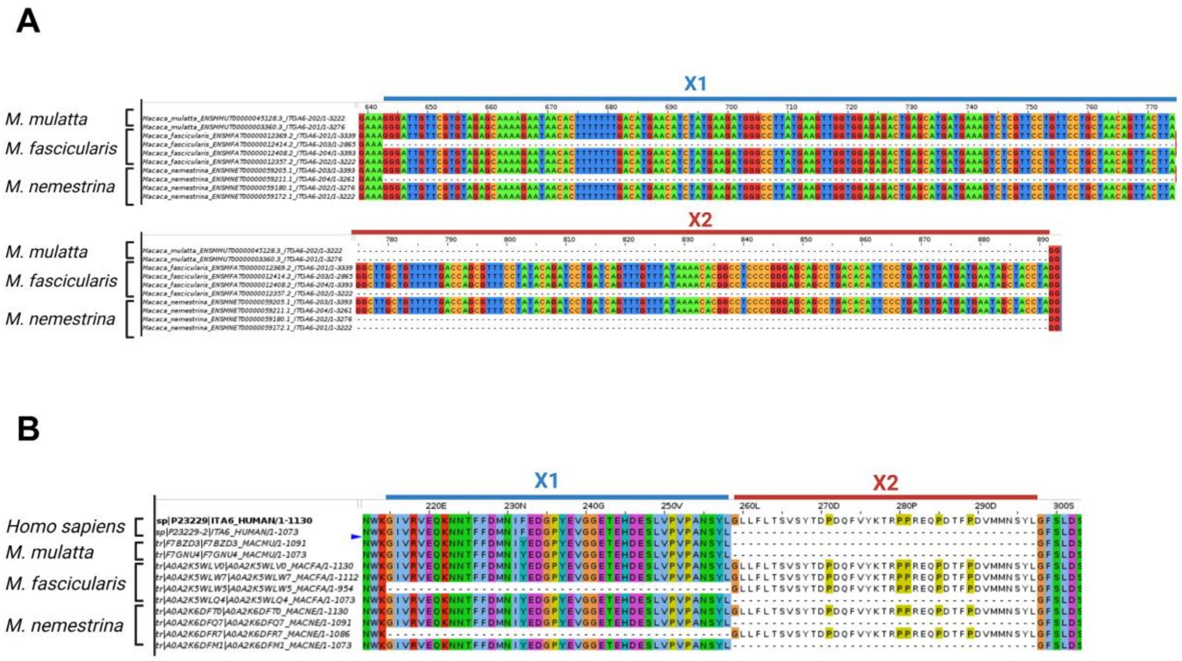

Figure S1. Nucleotide and protein sequence alignments of ITGA6 exons X1 and X2 in macaque species. (A) Nucleotide sequence alignment of exons X1 (blue) and X2 (red) from *Macaca mulatta*, *Macaca fascicularis*, and *Macaca nemestrina* ITGA6 transcripts. (B) Corresponding protein sequence alignment of the X1 and X2 regions from human (*Homo sapiens*) and the three macaque species.

Human ITGA6 Exon X1

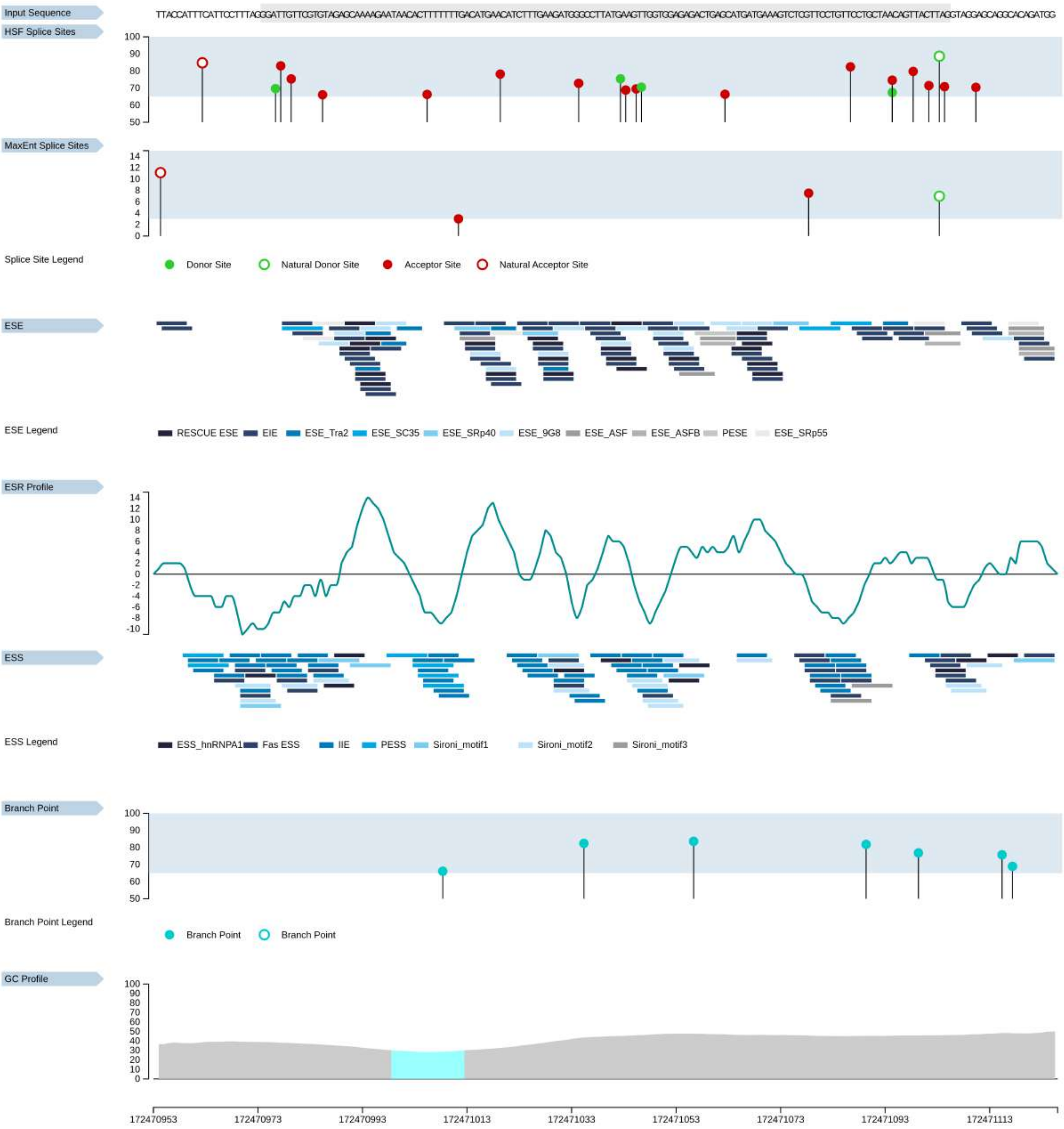

Human ITGA6 Exon X2

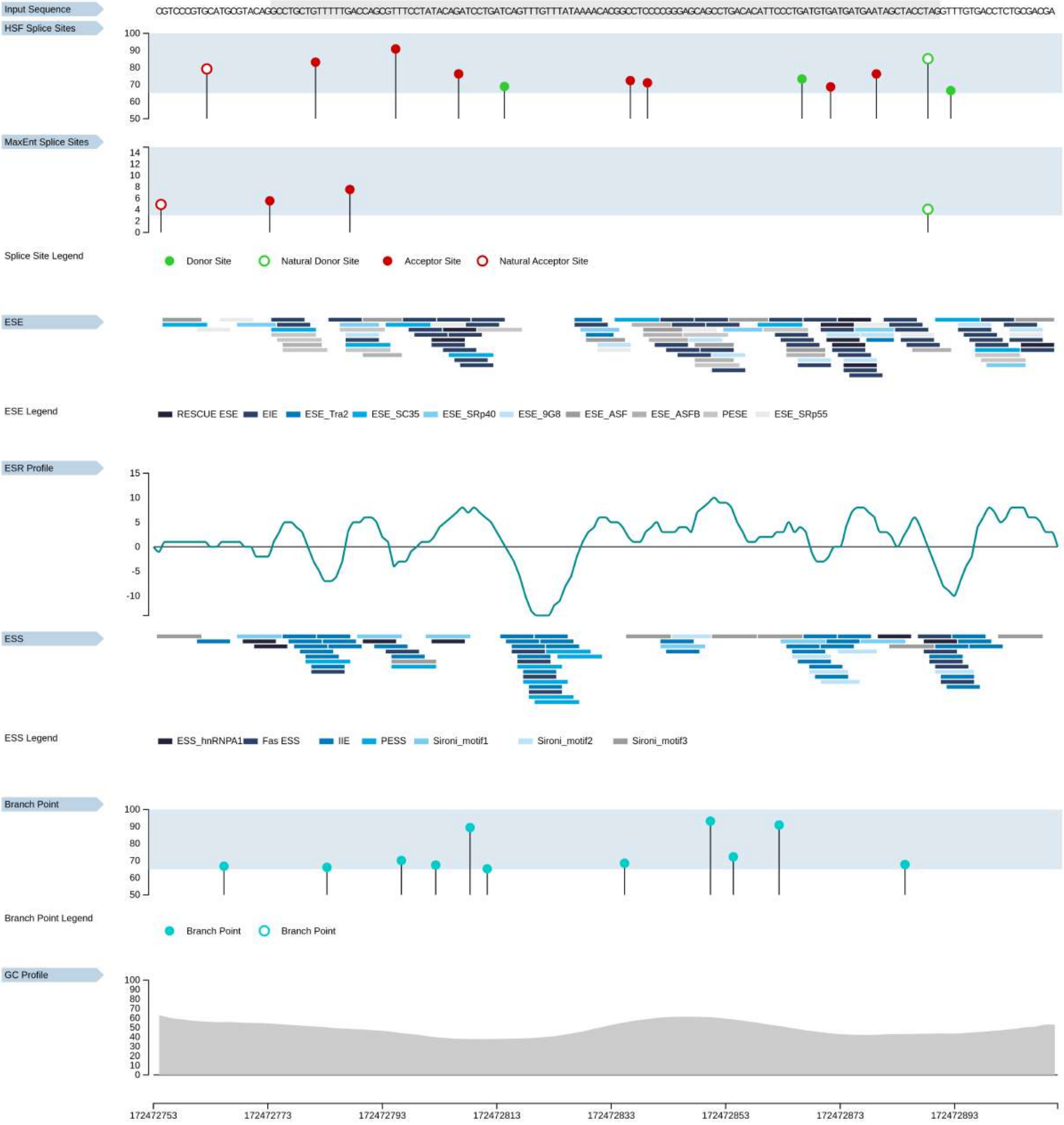

Human ITGA7 Exon X1

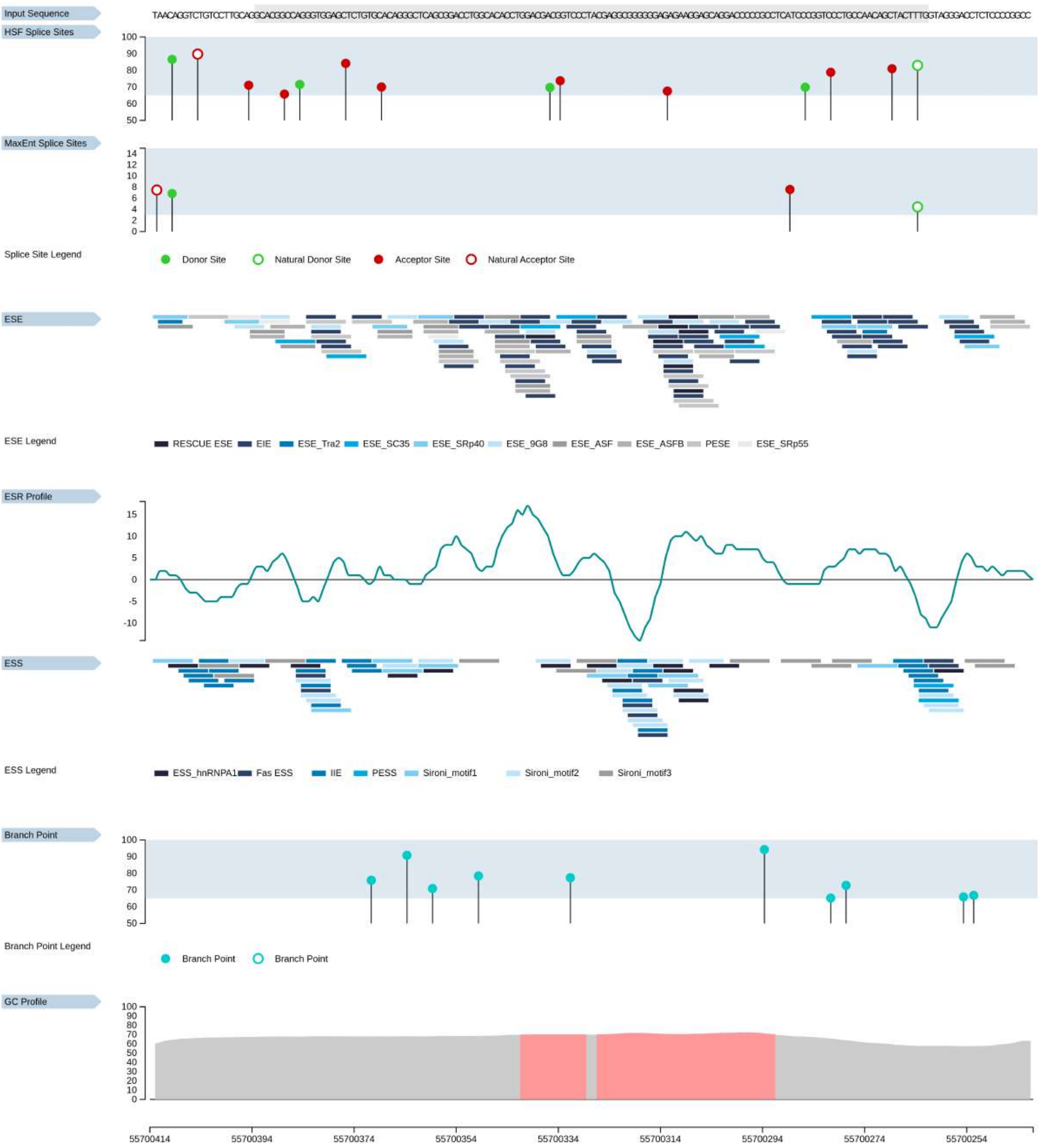

Human ITGA7 Exon X2

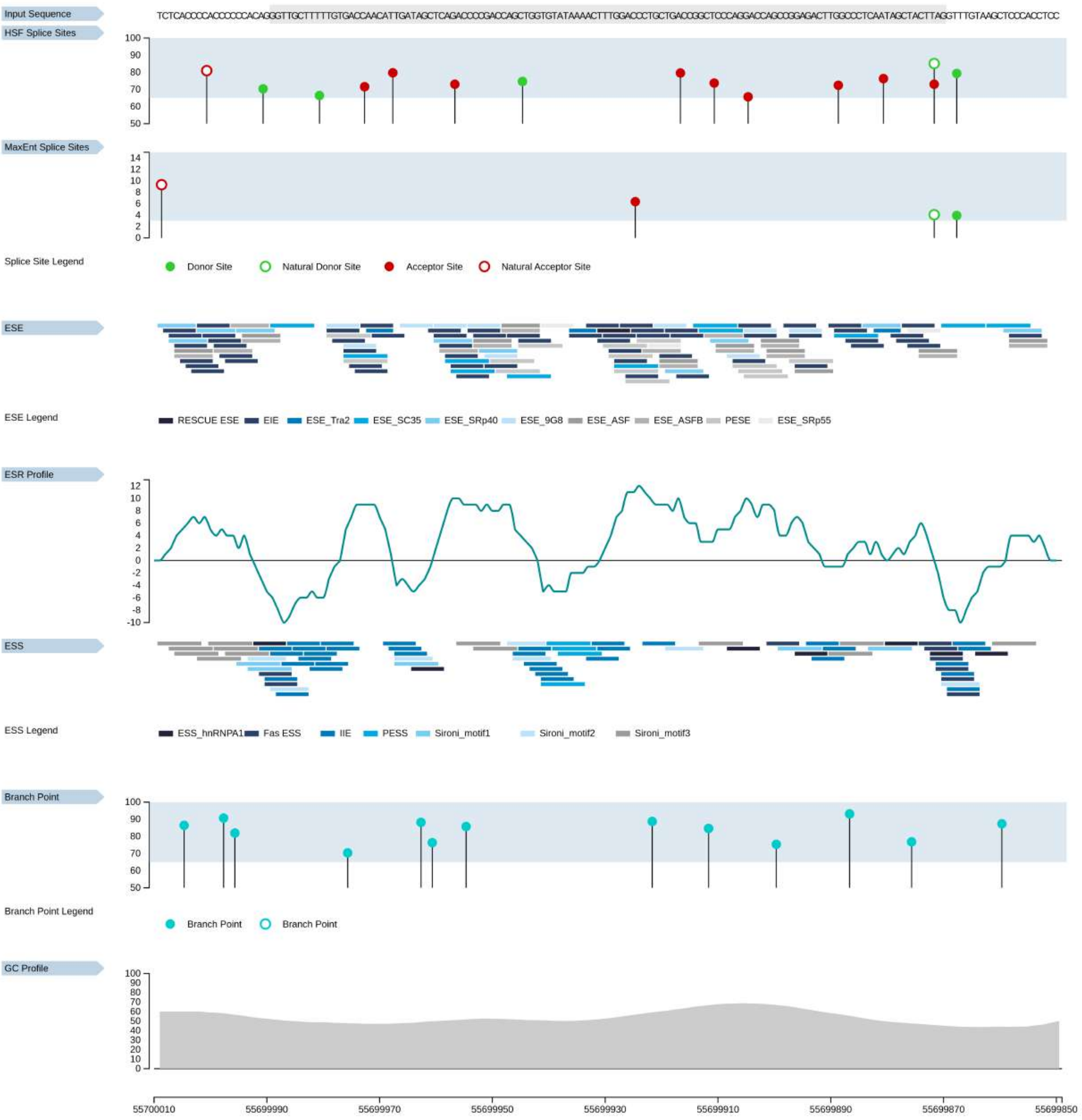

Figure S2. Human Splice Finder analysis of ITGA6 and ITGA7 X1 and X2 exons showing predicted splice donor/acceptor sites and exonic splicing enhancer (ESE) and silencer (ESS) motifs.

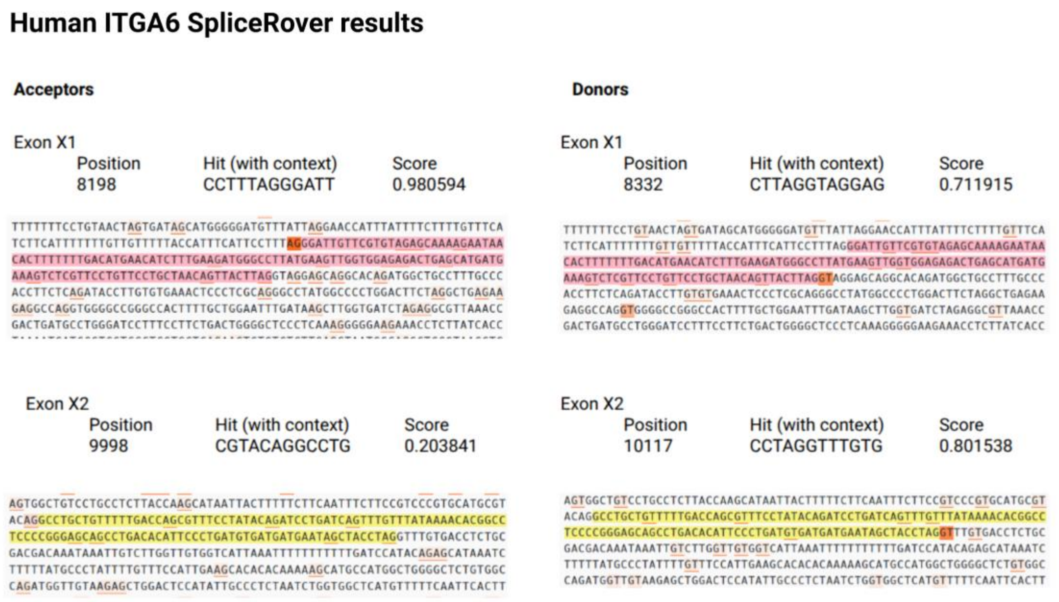

Figure S3. SpliceRover results of human ITGA6. Predicted acceptor (left) and donor (right) splice sites are shown for exons X1 and X2, with genomic positions, sequence context, and prediction scores.

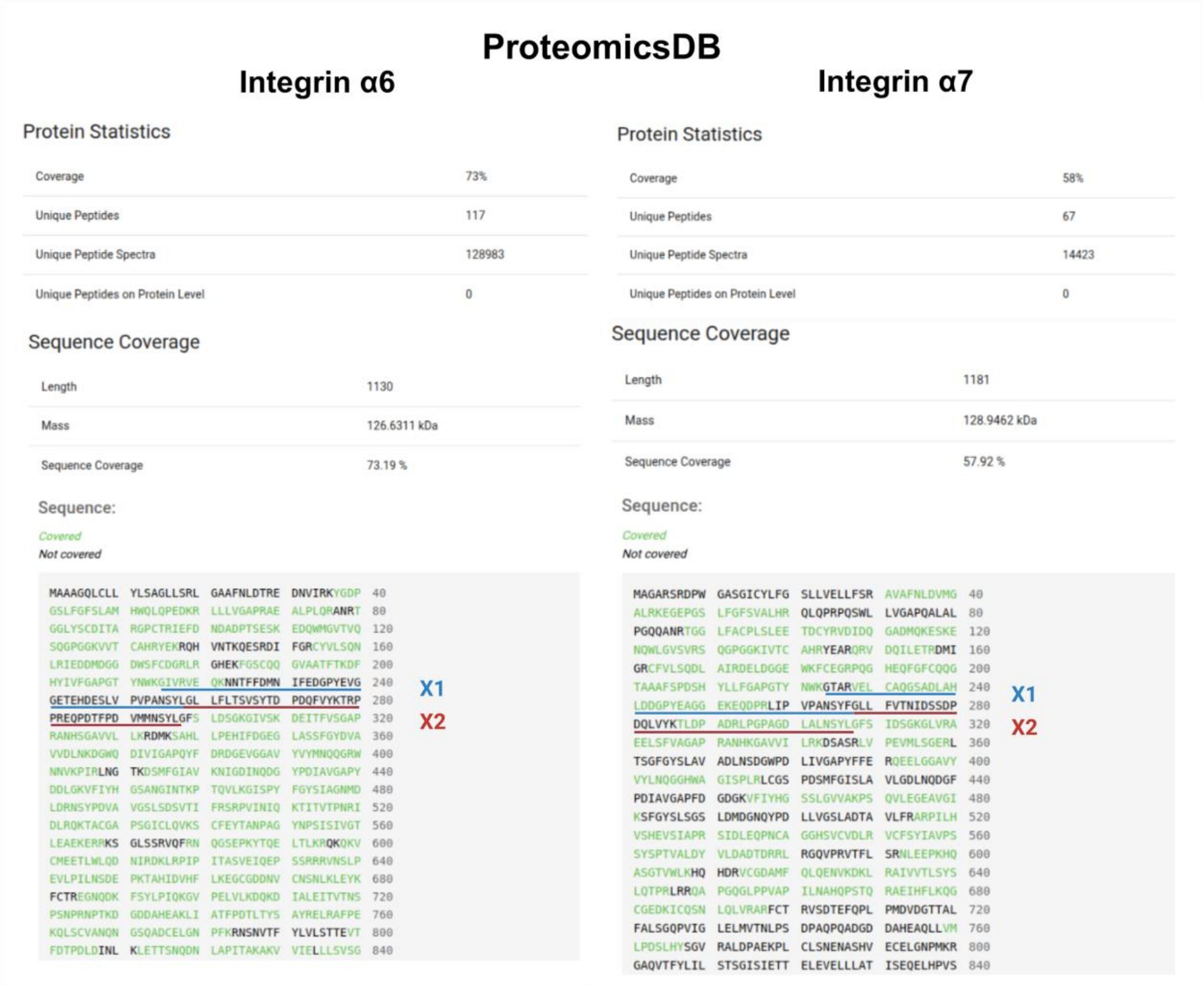

Figure S4. ProteomicsDB statistics and sequence coverage maps for human integrin α6 (left) and integrin α7 (right). Detected peptide sequences are highlighted in green, with undetected regions in black. The positions of exons X1 (blue) and X2 (red) are indicated within the protein sequences.

PeptideAtlas

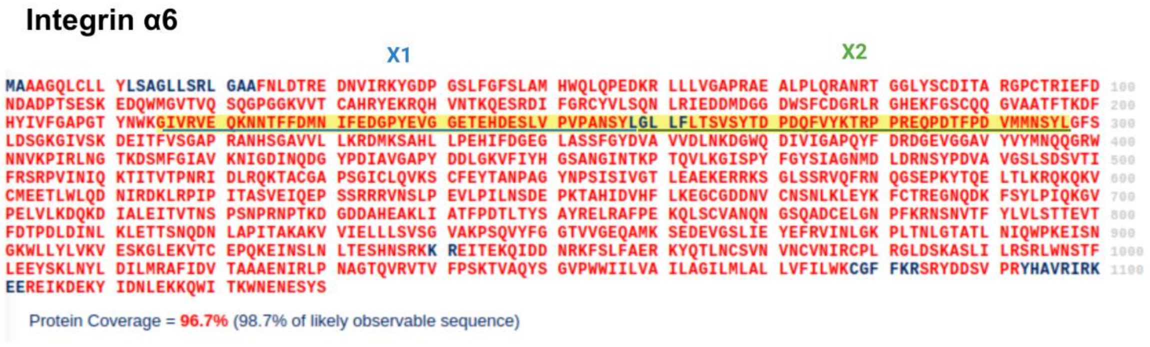

Figure S5. PeptideAtlas protein coverage map for integrin α6, with detected peptides highlighted in red and undetected residues in grey. The positions of exons X1 (blue) and X2 (green) are indicated within the sequence.

Supplementary tables

Table S1. List of reported variants within ITGA6 exons X1 and X2, including variant ID, genomic and residue positions, exon location, variant class, protein change, clinical significance, predicted consequence, known disease associations, allele frequency in gnomAD, and source databases.

Table S2. List of reported mutations within ITGA6 exons X1 and X2 from COSMIC, including residue position, mutation name, exon location, mutation ID, observed count, mutation type, affected tissues, and curation status.

Table S3. Length-normalized somatic variant burden in alternative exons X1/X2 of ITGA6 and ITGA7. COSMIC Variants counts unique somatic coding variants within the exon boundaries, COSMIC Variants/kb is the number of unique somatic coding variants in an exon, divided by the exon's length in base pairs, then multiplied by 1000 to express the rate per kilobase. Germline Pathogenic Variants lists ClinVar variants mapped to each exon. Exon length refers to coding bases only.
